# Prediction of Alzheimer's disease stages based on ResNet-Self-attention architecture with Bayesian optimization and best features selection

**DOI:** 10.3389/fncom.2024.1393849

**Published:** 2024-04-25

**Authors:** Nabeela Yaqoob, Muhammad Attique Khan, Saleha Masood, Hussain Mobarak Albarakati, Ameer Hamza, Fatimah Alhayan, Leila Jamel, Anum Masood

**Affiliations:** ^1^Department of Computer Science and Mathematics, Lebanese American University, Beirut, Lebanon; ^2^IRC for Finance and Digital Economy, King Fahd University of Petroleum and Minerals, Dhahran, Saudi Arabia; ^3^Department of Computer and Network Engineering, College of Computer and Information Systems, Umm Al-Qura University, Makkah, Saudi Arabia; ^4^Department of Information Systems, College of Computer and Information Sciences, Princess Nourah bint Abdulrahman University, Riyadh, Saudi Arabia; ^5^Department of Physics, Norwegian University of Science and Technology, Trondheim, Norway

**Keywords:** Alzheimer's disease, MRI, deep learning, self-attention, convolutional neural network, optimization, fuzzy entropy

## Abstract

Alzheimer's disease (AD) is a neurodegenerative illness that impairs cognition, function, and behavior by causing irreversible damage to multiple brain areas, including the hippocampus. The suffering of the patients and their family members will be lessened with an early diagnosis of AD. The automatic diagnosis technique is widely required due to the shortage of medical experts and eases the burden of medical staff. The automatic artificial intelligence (AI)-based computerized method can help experts achieve better diagnosis accuracy and precision rates. This study proposes a new automated framework for AD stage prediction based on the ResNet-Self architecture and Fuzzy Entropy-controlled Path-Finding Algorithm (FEcPFA). A data augmentation technique has been utilized to resolve the dataset imbalance issue. In the next step, we proposed a new deep-learning model based on the self-attention module. A ResNet-50 architecture is modified and connected with a self-attention block for important information extraction. The hyperparameters were optimized using Bayesian optimization (BO) and then utilized to train the model, which was subsequently employed for feature extraction. The self-attention extracted features were optimized using the proposed FEcPFA. The best features were selected using FEcPFA and passed to the machine learning classifiers for the final classification. The experimental process utilized a publicly available MRI dataset and achieved an improved accuracy of 99.9%. The results were compared with state-of-the-art (SOTA) techniques, demonstrating the improvement of the proposed framework in terms of accuracy and time efficiency.

## 1 Introduction

Dementia is the seventh-greatest root cause of mortality and the main reason for impairment and vulnerability in elderly individuals (Koul et al., 2023). It is a rapidly spreading disorder among the elderly population, becoming increasingly common over the last decade (Sisodia et al., 2023). Dementia greatly impairs intellectual performance, interfering with daily tasks and interpersonal interactions (Nagdee, 2011). Alzheimer's disease (AD) is an inseparable subclass of dementia that can cause memory loss in a person (Mahmud et al., 2024). An individual affected by AD may struggle to recognize family members and experience difficulties in remembering daily activities. Moreover, it can cause ultimately lead to the death of the patient (Mohammad and Al Ahmadi, 2023). Due to these worse health conditions, it is also referred to as a progressive neurodegenerative disease. It affects behavioral functions, thinking abilities, decision-making, and language skills, often leading to memory loss in older people (Kellar and Craft, 2020).

Brain cell alteration may occur a decade or more before clinical signs appear. In the beginning, patients with AD experience unnoticed changes in their brains (Jansi et al., 2023). Throughout the early AD stage, the brain undergoes destructive transformations, including ectopic protein deposition that produces amyloid plaques and tau tangles. Neurons that were once fully functional cease to function properly, losing connections to other neurons and eventually undergoing cell death (Hoozemans et al., 2006). Several additional intricate alterations in the brain can also lead to Alzheimer's (Kasula, 2023). The hippocampus and entorhinal cortex, critical for cognitive control, seem to be the initial regions of impairment (Shrager et al., 2008). Furthermore, the signs of AD begin to manifest when nerve cells (neurons) in certain areas of the brain gradually shrink and eventually become destroyed or damaged (Khalid et al., 2023). In the final phase of AD, damage becomes widespread, and a large amount of brain tissue is destroyed (Bloniecki Kallio, 2002; Carle, 2022).

AD is such a serious brain disease that it can result in a patient's death if not effectively treated (Gómez-Isla and Frosch, 2022). To overcome this disease, patients need good care, regular exercise, and some memory-sharpening activities as there is currently no specific medication for AD (Shamrat et al., 2023). In recent years, a significant increase has been observed in AD (Mirzaei and Adeli, 2022; Stevenson-Hoare et al., 2023). The number of deaths from Alzheimer's disease in 2020 increased by 15,925 compared to the 5 years before 2023, and 44,729 more deaths were recorded for all dementias, including Alzheimer's disease (Chua, 2023). Traditional machine learning (ML) techniques such as pre-processing (Wen et al., 2020), feature extraction (Rathore et al., 2017), feature selection (Balaji et al., 2023), feature fusion (Jia and Lao, 2022), and classification (Tanveer et al., 2020) have been employed by researchers as a four-step channel in the past few years. Classification is the bottommost step in which each object accredits a label, in either a supervised or unsupervised ML technique (Bondi et al., 2017). Deep learning (DL) (Shaukat et al., 2022) is a subtype of machine learning that falls under the umbrella of artificial intelligence, but DL is way more vigorous and flexible in comparison with ML (Fabrizio et al., 2021). Techniques such as shallow CNN (Marwa et al., 2023), DNN (Hazarika et al., 2023), MultiAz-Net (Ismail et al., 2023), hybridized DL method (Hashmi, 2024), and RVFL (Goel et al., 2023) have been used in recent years, but these techniques yield low accuracy as compared to our proposed model (Shamrat et al., 2023).

### 1.1 Major challenges and gaps

Recent advances in ML and DL have opened up new avenues for assessing AD, but researchers are still grappling with the diagnosis of the disease (Shamrat et al., 2023). Few of them are related to insufficient and unbalanced datasets. Furthermore, major problems with AD patients are the complexity, diversity, and complicated neurobiological underlying AD (Dhakhinamoorthy et al., 2023). Architectural variation in scans is another main challenge to diagnosing and detecting AD. However, the influence of these challenges may vary from patient to patient. This research will focus on AD stages for classification using deep learning and feature optimization techniques.

### 1.2 Major contributions

The main contributions of this study are as follows:

- A fine-tuned ResNet-50 architecture has been modified by adding a self-attention layer and trained from scratch for feature extraction.
- Hyperparameters of the trained model are initialized using an optimization technique named Bayesian optimization.
- Improved the extracted self-attention features using an improved pathfinder optimization named the Fuzzy entropy-controlled path-finding algorithm (FEcPFA). The optimization algorithm selects the best features and improves the efficiency.
- The optimized selected features are finally classified using machine learning to classify the stages of AD.

This article is organized as follows: Section 2 reviews ML and DL techniques that have been applied to Alzheimer's disease, and Section 3 provides a comprehensive description of the datasets. The testing outcomes are shown in detail in Section 4. Section 5 summarizes our findings, and Section 6 discusses future work.

## 2 Related work

Due to the brain's intricacy, classifying AD is difficult (Dhakhinamoorthy et al., 2023). Thus, researchers are improving medical image processing to identify AD correctly. This section presents relevant literature in the domain of AD detection and diagnosis, which focuses primarily on classification techniques based on deep learning for MRI tissue structure analysis (Mohi et al., 2023). The deep belief network (DBN) was utilized by AI-Atroshi et al. (2022) to extract feature vectors from detected speech samples, which has an output accuracy of 90.2%. Shankar et al. (2022) used HAAR-based object identification techniques because they are more suitable with discriminant attributes and generated 37 spatial pieces of information from seven characteristics that produced 94.1% accuracy on the dataset taken from ADNI. To aid in the initial diagnosis of AD (FDN-ADNet), Sharma et al. (2022) used a DL network for all-level feature extraction from extracted sagittal plane slices of 3D MRI scans and a fuzzy hyperplane-oriented FLS-TWSVM for the classification of the retrieved features, which generated 97.29% accuracy on the publicly available ADNI dataset.

Albright (2019) presented the all-pairs pre-processing algorithm to train the model. For this experiment, setting data were taken from ADNI and divided into three datasets, i.e., LB1, LB2, and LB3, with an mAUC of 0.866. The 3D-CNN networks by Soliman et al. (2022) predicted AD. It learned basic traits that catch AD indicators to identify brains with Alzheimer's disease from healthy and normal brains using MRI scans. ADNI provided 3,013 photographs with 96.5% training accuracy and 80.6% tested accuracy. Samhan et al. (2022) adopted CNNs, VGG16, Adam, activation, and softmax optimizers. The Kaggle dataset of 10,432 images yielded 100% training accuracy, 0.0012 training loss, 97% validation accuracy, and 0.0832 verifying loss. Jo et al. (2022) proposed a unique deep learning-based genome-wide approach called SWAT-CNN that found SNPs associated with AD and a classification model for AD. It may be useful for a variety of biomedical applications and was tested on the GWAS dataset by the AD Neuroimaging Initiative (ADNI).

Zhang et al. (2022) adopted CNN models of various designs and capacities and assessed them thoroughly. The most appropriate model was then applied for AD diagnosis. To increase the transparency of the model, an explanation heatmap was produced for AD vs. cognitive normal (CN) classification tasks and pMCI vs. sMCI using two publicly available datasets. Interestingly, the study found that a moderately sized model could outperform one with the largest capacity. Ghazal et al. (2022) proposed the system named ADDLTA, in which the transfer learning (TL) approach was used in conjunction with brain medical resonance imaging (MRI) to classify the image into four categories: mildly demented (MD), moderately demented (MD), non-demented (ND), and very mildly demented (VMD), which gave 91.70% accuracy on simulation results based on the publicly assessable dataset by the Kaggle repository.

Shanmugam et al. (2022) focused on detecting different phases of cognitive impairment and AD in the early stages by utilizing TL in neuroimaging. GoogLeNet, AlexNet, and ResNet-18 were three pre-trained models adopted for classification, giving an accuracy of 96.39, 94.08, and 97.51%, respectively, on the ADNI dataset. Prasath and Sumathi (2024) suggested a compact architecture by merging two models, LeNet and AlexNet, that outperform DenseNet. Three parallel tiny filters (1 × 1, 3 × 3, and 5 × 5) replaced the convolution levels to recover key features that achieved 93.58% accuracy on the dataset taken from ADNI. Sorour et al. (2024) proposed a system for the automated diagnosis of Alzheimer's disease that integrates multiple customized deep-learning models to provide an objective evaluation. The very first methodology addresses AD diseases using SVM and KNN. The second approach combines rs-fMRI datasets from the ADNI repository with modified AlexNet and Inception blocks. This architecture gave 96.61% accuracy. A new optimized ensemble-based DNN learning model called MultiAz-Net is used by Ismail et al. (2023) with diverse PET and MRI data to identify AD. The Multi-Objective Grasshopper Optimization Algorithm (MOGOA) optimizes MultiAz-Net layers, which produced 92.3% accuracy on the ADNI dataset. Balaji et al. (2023) suggested a DL approach to detect AD in its initial stages using multimodal imaging and the LSTM algorithm, combining MRI, PET, and traditional neuropsychological examination results. The suggested technique adjusted the learning weights to improve accuracy and employed Adam's optimization. The proposed architecture achieved 98.5% accuracy on 512 MRI and 112 PET scans.

## 3 Materials and methods

This section provides a comprehensive exposition of the experimental dataset and methodologies employed within. It elucidates the specifics of the experiments, including the nature of the dataset utilized and the methodologies adopted.

### 3.1 Dataset

**Figure 1:**
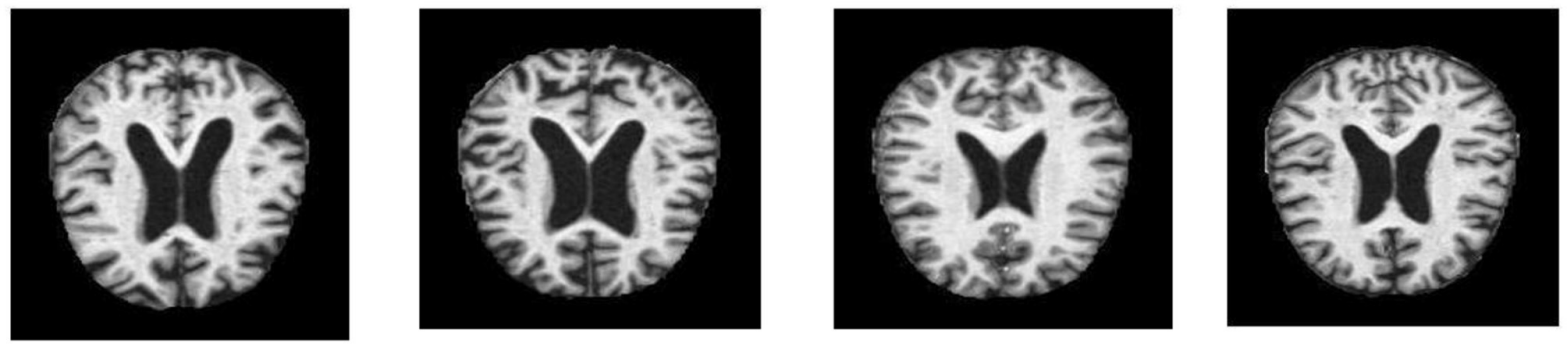
Classes of Alzheimer's disease for the classification.

A well-characterized repository has a significant role in the performance evaluation of a diagnosis system. In this experiment, a dataset was obtained from Kaggle. This dataset, known as Alzheimer's disease, consists of specimens of anonymously affected individuals with MRI scans and their appropriate class label details. This multiclass dataset contains four distinct classes and offers many different views, comprising over 5,000 MRI images. The four classes are shown in Figure 1: mildly demented (Shanmugam et al., 2022), moderately demented (Prasath and Sumathi, 2024), non-demented (Sorour et al., 2024), and very mildly demented (Ismail et al., 2023). A brief explanation of the four classes of AD is given in Table 1 for testing and training purposes. The data were imbalanced in each class. Each class consisted of a different number of images.

**Table 1:** Description of AD classes dataset.

**References**	**Classes**	**Description**	**No of images**
Shanmugam et al. (2022)	Mild demented	People may become socially withdrawn, and noticeable changes occur in their moods and personality. People may find it hard to remember the faces, people they met a long time ago, and recent events. Individuals do not recall what they are saying, cannot find their way to their desired location, and have lost focus and work abilities.	896
Prasath and Sumathi (2024)	Moderate demented	In this phase, the affected person requires help to do their routine work. Inability to recall important information, such as name of close relatives, home location, time, and date; however, the person knows their name and family member's names. The person lacks sensibility, forgets previous work, and struggles to keep track of finances and daily expenses while living alone.	3,200
Sorour et al. (2024)	Non-demented	It usually occurs in elderly persons. People may face difficulty in conversation and gradually memory loss.	64
Ismail et al. (2023)	Very mild demented	The person may find it hard to adjust to a new environment and experience apathy and repetition. Affected persons cannot complete the task. There seems to be low memory loss in this stage. Individuals may forget the names of people who lived with them.	2,240

**Figure 2:**
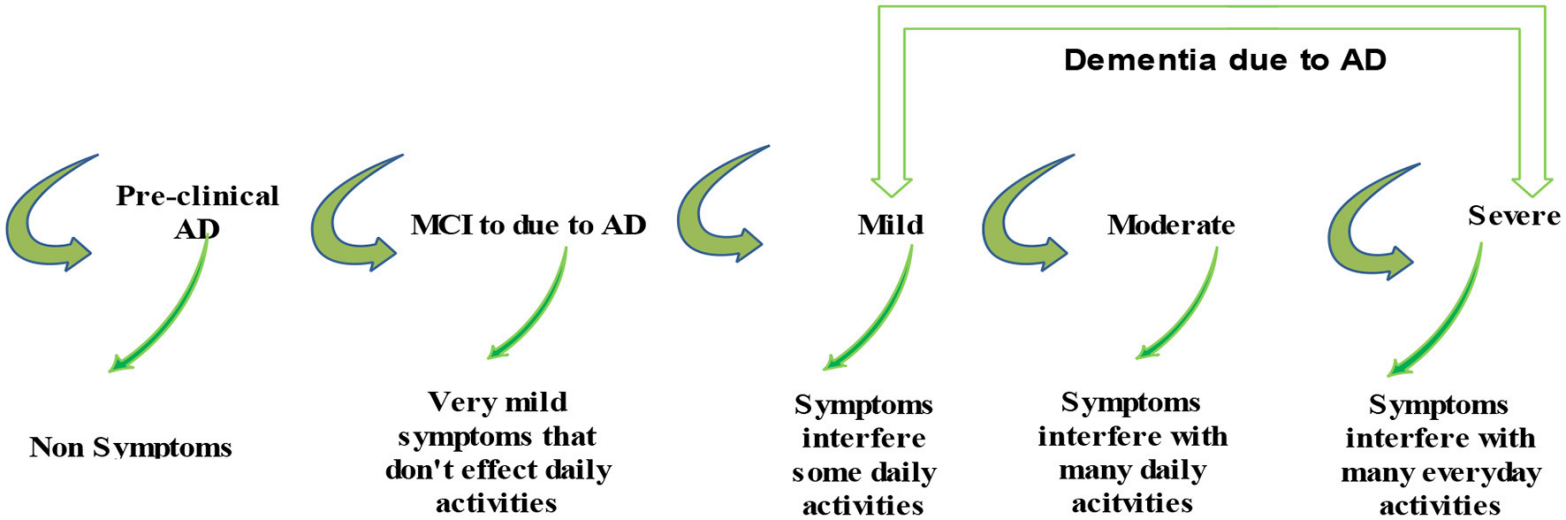
Description of AD stages.

These datasets are the most prominent and effective for this publicly available domain. The major aim of this study is to yield high accuracy. Original MRI scans and augmented image distribution were utilized in the training and testing of the experiment. Mild demented contained 896 images; moderate demented contained 64 images; non-demented comprised 3,200 images; and very mild had 2,240 images. After the augmentation, we took 2,000 images from each class for further proceedings. Figure 2 illustrates the AD stages with a brief description. Moreover, an image description that lists the number of classes and augmented images utilized in this study is found in Table 2.

**Table 2:** Dataset image description.

**S#**	**Dataset**	**Number of classes**	**Total images**	**Augmented images**	**Training/testing**
1	Alzheimer's disease	Mild demented	896	3,200	3,200/2 = 1,600
2		Moderate demented	64	3,200	3,200/2 = 1,600
3		Non-demented	3,200	3,200	3,200/2 = 1,600
4		Very mild demented	2,240	3,200	3,200/2 = 1,600

### 3.2 Proposed methodology

**Figure 3:**
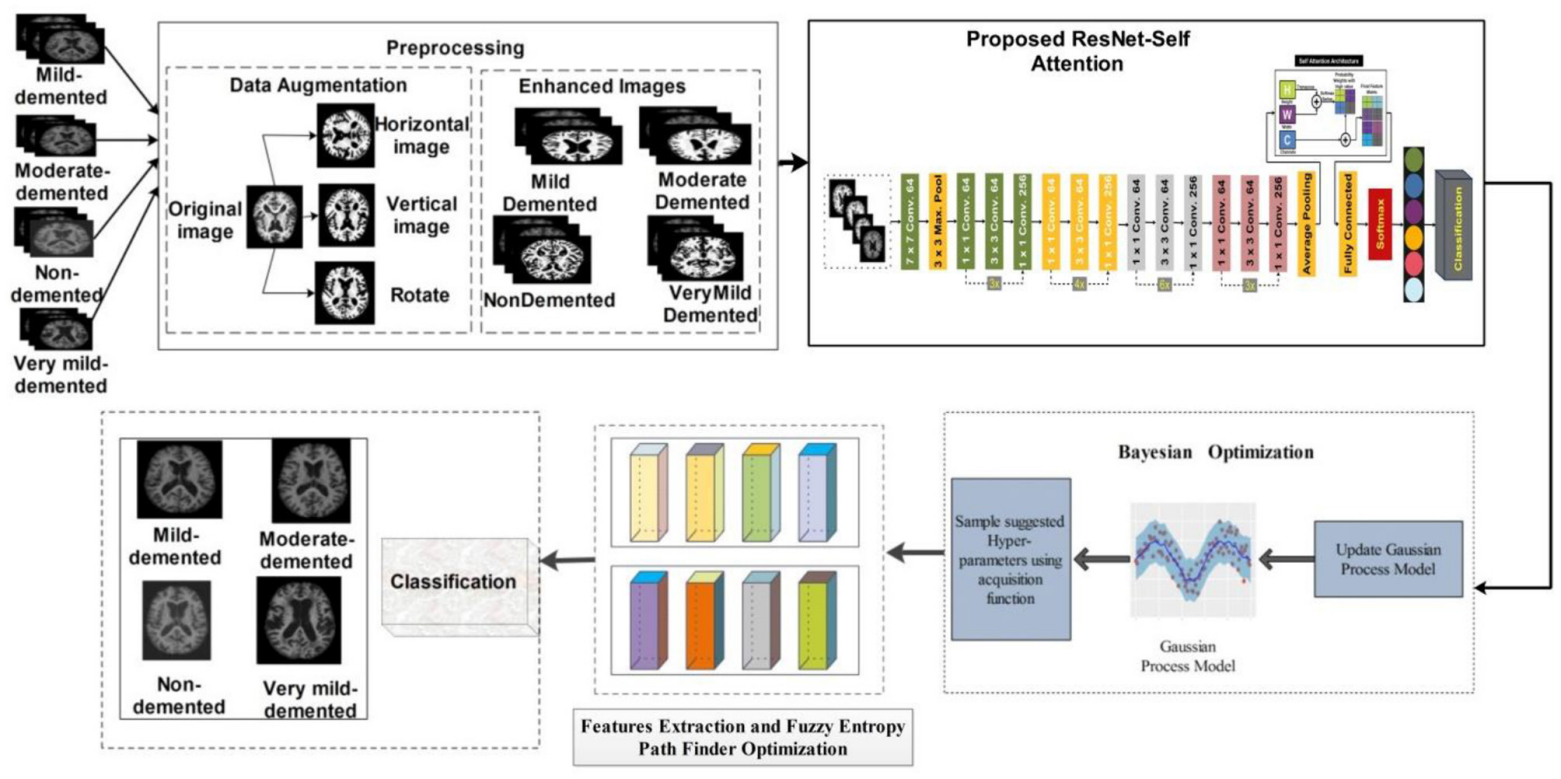
Proposed model of classification of AD stages.

Our proposed study presents a deep learning-based methodology for classifying AD grades. First, the dataset was taken from Kaggle, a public repository. The data were unbalanced in each class, so different augmentation techniques were applied. The data have been enhanced by applying different enhancement methods. After the enhancement, we fine-tuned the ResNet-50 model and added Self-Attention layers. The modified model is trained on the augmented dataset and extracted deep features from the self-attention layer. The features are extracted from the self-attention layer. Bayesian optimization is employed for the selection of hyperparameters, instead of manual initialization. Moreover, PFA is utilized to select the optimal features. In the final stage, KNN, NN, and SVM classifiers are used to classify AD stages. The proposed model is represented in Figure 3.

#### 3.2.1 Data augmentation

Augmentation is creating modified image variants from an existing image dataset to improve its variety artificially. Images are nothing more than a 2D collection of numbers for a computer. These numbers indicate intensity values, which may be modified to produce new, enhanced images. The primary goal of augmentation is to maintain parity among each group. It improved the outcomes and made them more precise and effective. In most cases, it was only useful for very small data sets. Images may be flipped horizontally, vertically, or rotated using this method. Both of these techniques expand the quantity of the dataset by producing images that have been flipped at various angles.

##### 3.2.1.1 Horizontal flip

Complete rows and columns of image pixels are set aside horizontally. If the image on the right is flipped, the outcome will be on the left. The mathematical representation for the horizontal flip is shown by Equation 1.


(1) $$\begin{array}{l} H_{F\;}\left(-x,\;y\right)=\;H_{O}\;\left(x,y\right) \end{array}$$

The given formula illustrates the horizontal flip of an image scan. HF shows the flipping function, while H_O_ represents the real image. The first half (*x, y*) displays the actual image, while quadrant two (−*x, y*) displays the replica image. Therefore, the unedited version of the image resides within the first quarter, which is the right side, and after horizontal flipping, the image has been flipped to the second phase, which is the left side.

##### 3.2.1.2 Vertical flip

Complete rows and columns of image pixels are set aside vertically. When an image is now displayed in the upward position and flipped, the resulting image will be displayed in the downward motion. The mathematical representation for the vertical Flip is shown below:


(2) $$\begin{array}{l} H_{v\;}\left(x,\;-y\right)=H_{O}\left(x,y\right) \end{array}$$

The given formula illustrates the vertical flip of an image scan. HV shows the flipping function, while H_O_ represents the real image.

The first half lies in (*x, y*) which displays the actual image, while the third quadrant third (*x*, −*y*) displays the replica image. Therefore, Equation (2) demonstrates that the initial image resides in the first half on the right side. When the vertical flip is enforced, the image goes to the third half, which is in a downward direction. In short, it flipped the image along with the *X*-axis.

##### 3.2.1.3 Rotate flip

A 3D graphic item is flipped by rotating it. The following is a mathematical representation by Equation 3.


(3) $$g_{\left(i,j\right)}^{90^{{}^{\circ}}}=\left[\begin{array}{cc} \cos90^{{}^{\circ}} & -sin90^{{}^{\circ}}\\ \sin90^{{}^{\circ}} & \cos90^{{}^{\circ}} \end{array}\right]\left[\begin{array}{c} g_{i}\\ g_{j} \end{array}\right]$$

**Figure 4:**
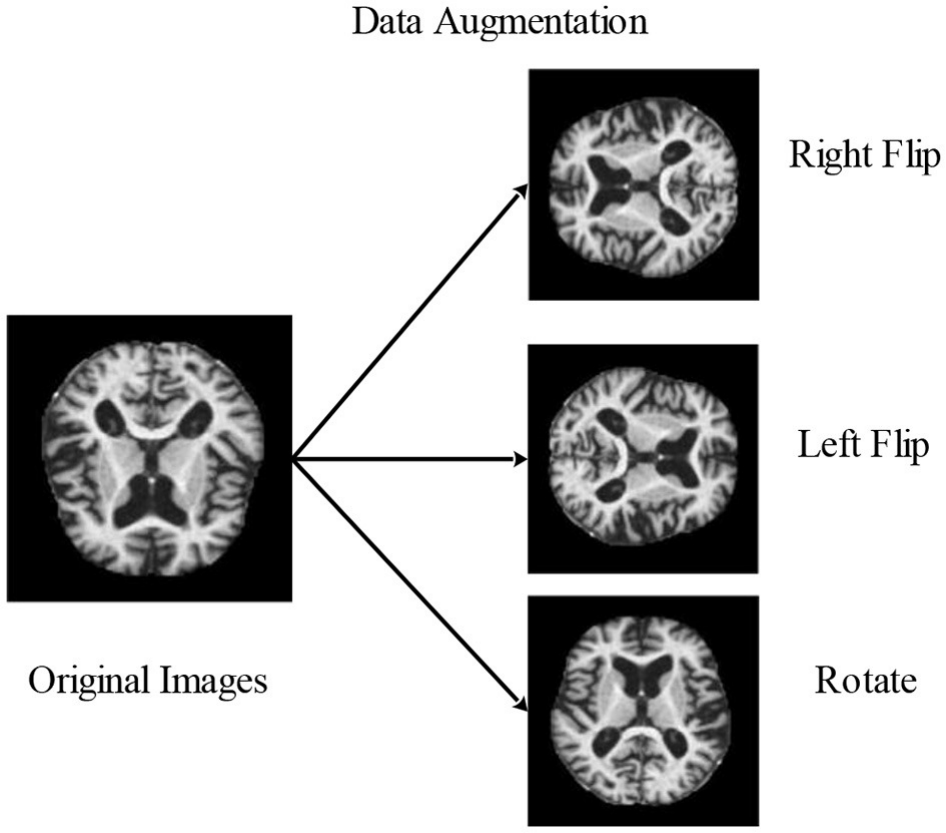
Visual illustration of image augmentation using mathematical techniques.

Consequently, image (I) is rotated by angle degrees counterclockwise around its center. To rotate the image counterclockwise, input a negative angle value, then (imrotate) will extend the resultant image (J) to encompass the entire rotated image. The methods for data augmentation used in the experiment are shown in Figure 4.

#### 3.2.2 Contrast enhancement

Increasing contrast is one of the most important and useful techniques for improving the essential elements of an image. Normally, raw images contain noise, distortion, and low contrast that lower the image quality, which sometimes causes the loss of useful information (Perumal and Velmurugan, 2018). Contrast enhancement improves the image qualities for further processing. More relevant characteristics may be extracted from the improved photographs for the classification stage than from the input image. The datasets chosen in this study have poor-quality images with low contrast levels. Due to this issue, we could end up incorrectly categorizing things. Contrast enhancement is further divided into two main groups, i.e., the spatial and frequency domains, such as morphological enhancement, histogram equalization, contrast stretching, contrast slicing, and some contrast enhancement. In our proposed experiment, two types of contrast enhancement have been adopted, one by one. First, a fast local Laplacian filter is applied to the augmented dataset. After this, a top–bottom hat filter is applied to the enhanced dataset to get better-quality MRI scans.

##### 3.2.2.1 Fast local Laplacian filter

There are two main functions of FLLF. The first is applied to the raw images to boost the boundary detail and reduce the noise artifacts. The second is that the images are transformed from the RGB color system to the YUV color space to isolate the Y factor. Multiscale adjustments are crucial to photo editing but are especially vulnerable to halos. Advanced edge-aware algorithms and careful parametric adjustments are needed to get outcomes without artifacts. These deficiencies were subsequently remedied through local Laplacian filters. These filters use typical Laplacian pyramids to generate a wide variety of effects. However, these filters are time-consuming, and their link to other methods is obscure.

##### 3.2.2.2 Top–bottom hat filter

**Figure 5:**
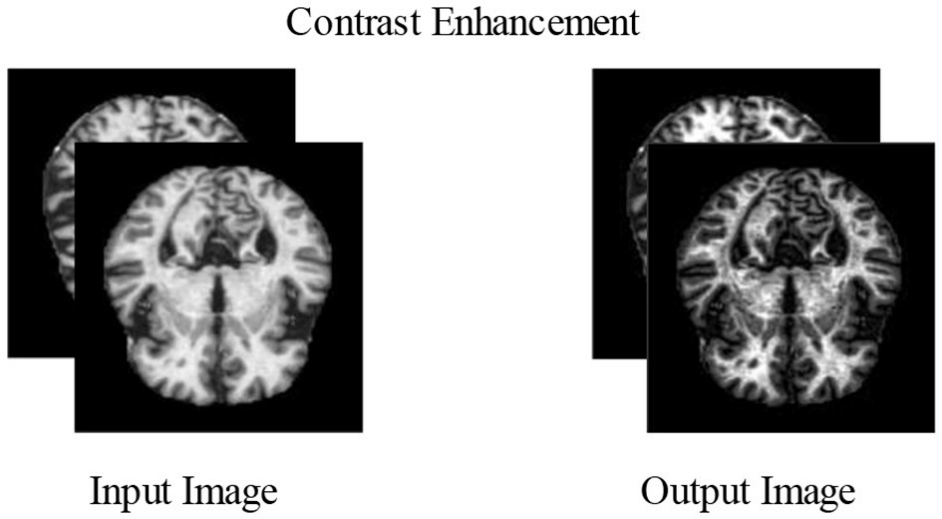
Visual illustration of contrast enhancement.

In this filter, the top-hat part is employed for objects with a light color on a darker backdrop, whereas the bottom-hat part is utilized for images with a dark color on a light background. The correction of the effects of non-lighting is a key purpose of the top-hat modification. When the shade is evident in an image, this filtering technique can effectively highlight the information in the image. The methods for contrast enhancement are visually presented in Figure 5.

#### 3.2.3 Bayesian optimization

Bayesian optimization incorporates Bayes' theorem to guide the search as a method for minimizing or maximizing an optimization technique. This method can be very helpful for optimization algorithms that are difficult to evaluate due to their complexity, noise, or cost. BO differs from other methods in that it considers previous parameter data by changing the baseline using Gaussian progress (GP). Additionally, BO has minimal iterations and a rapid convergence time. The BO approach may also eliminate local optimum in non-convex optimization circumstances. BO is a perfect pick for optimizing HPs due to its high convergence and resilience. All hyperparameters must be tuned to gain classification precision while utilizing DL architectures. The choice of hyperparameters substantially affects the accuracy and precision of the prediction. When optimizing hyperparameters, the objective is to choose the values that provide the highest quality validation findings. Hyperparameter optimization is written mathematically by Equation 4.


(4) $$\begin{array}{l} x^{*}=argminf\left(x\right) \end{array}$$

where f(x) shows the cost-minimizing objective score for evaluating hyperparameter optimization relative to the validation set, and x is the set of hyperparameters whose values lie in that range. Training takes longer and is extremely difficult to do by hand with DNN models with numerous hyperparameters. ML and simulations employ BO. FFNN designs alter hyperparameters in CV-based techniques to enhance the performance of the mode network. Optimizing several parameters is faster by using it.

**Figure 6:**
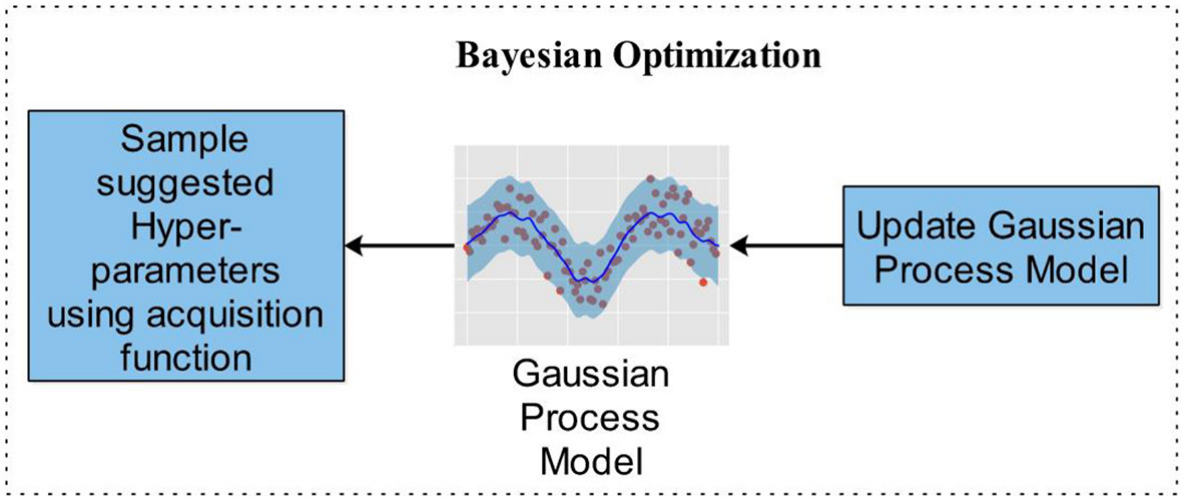
Bayesian optimization workflow for hyperparameters selection.

In contrast to other methods, BO updates the prior with Gaussian progress to adjust for past parameter values (GP). Additionally, BO converges quickly and with a small number of iterations. When addressing non-convex optimization problems, the BO approach may be able to sidestep localized optimality. BO is a great option for optimizing HPs due to its high convergence and resilience. The stopping condition of the BO algorithm is based on MaxTime. The BO algorithm stops when it reaches the MaxTime, which is 54,000 s. This time is approximately equivalent to 15 h. In this study, we utilized BO along with DCNN, which fine-tuned the hyperparameters to generate the lowest error rate with optimal results in an architecture. Optimizing parameters such as L2Regularization, Section Depth, Momentum, and Learning Rate have been used in this study, shown along with their ranges in Table 3, which represents the Bayesian optimization workflow. Figure 6 illustrates the BO.

**Table 3:** Hyperparameter range of BO.

**Hyperparameters**	**Ranges**
L2Regularization	(1e^−10^, 1e^−2^)
Section depth	(1, 3)
Momentum	(0.7, 0.98)
Learning rate	(0.0001, 1)

#### 3.2.4 Deep transfer learning

**Figure 7:**
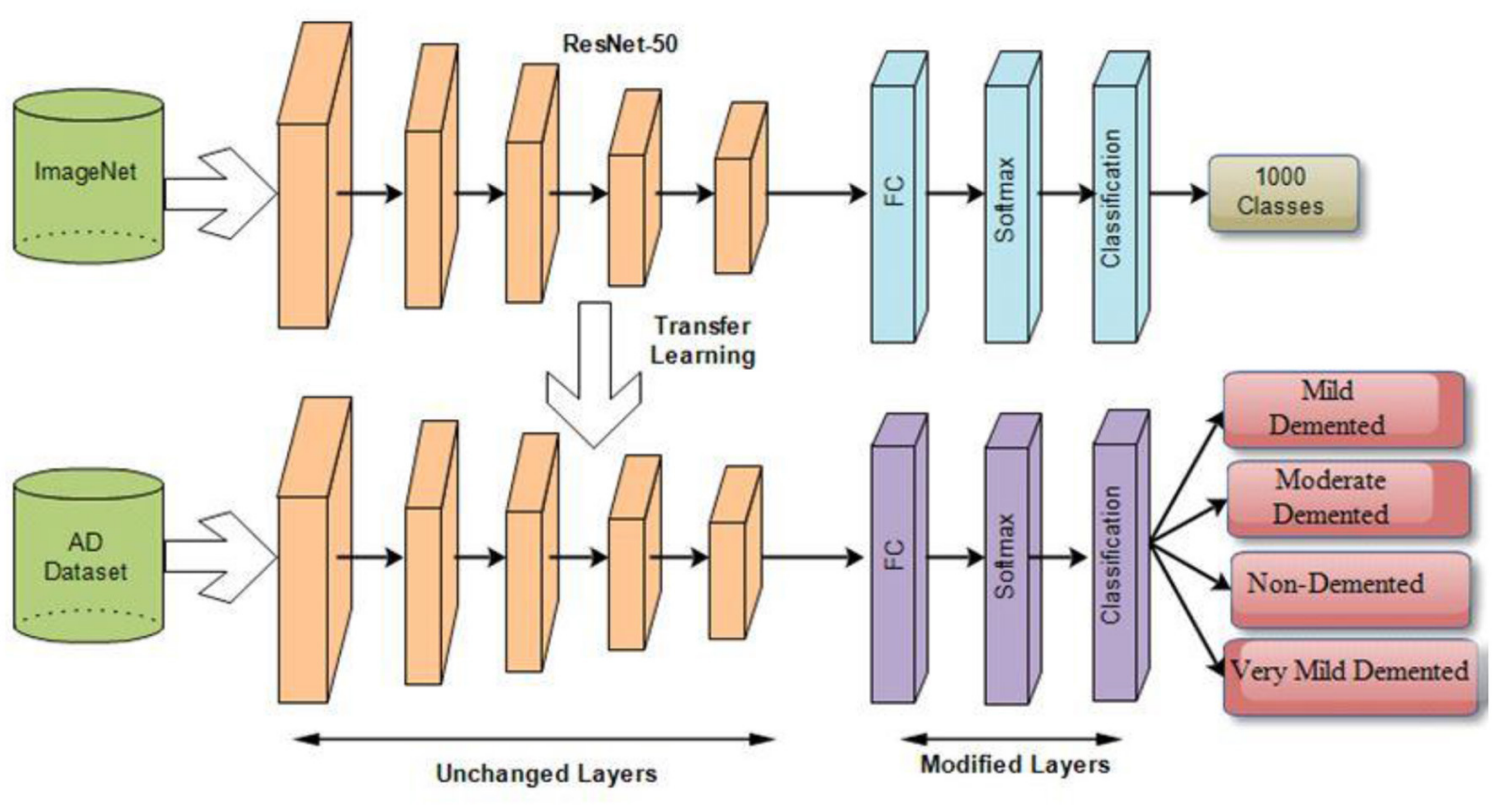
Deep transfer learning architecture for classification of AD stages.

Transfer learning is applying a learned model to a different situation. The fact that it has the potential to train deep neural networks on very little training data has recently made it more famous in deep learning. Deep transfer learning is becoming more prominent in handling image classification issues as it is feasible to use built-in CNNs on publicly available datasets such as ImageNet to achieve top classification accuracy in several application domains. After transfer learning (TL), the framework is fine-tuned (FT) to relearn all FE and C. FT is performed by initializing feature extraction parameters and ImageNet weights, and classification parameters are updated along with TL weights. Figure 7 illustrates the deep transfer learning workflow.

#### 3.2.5 Proposed ResNet-Self architecture

In this study, we proposed a modified ResNet-50 architecture based on the self-attention module named ResNet-Self. Initially, we consider the ResNet-50 architecture based on the residual blocks. In this network, 48 convolutional layers have been originally added, along with one max-pooling layer, one average pooling layer, and one fully connected layer. The residual blocks added in this network contain skip connections. In this network, bottleneck filters are applied, such as 1 × 1, which reduces the number of parameters. The depth size of this model is originally between 64- and 2,048, and filter sizes of 3 × 3. Moreover, the stride is used 2 out of the residual blocks, and in the residual blocks, 1 stride is employed. The average pooling layer has been added at the end of this model for the features extraction that followed the fully connected and softmax layers. The initial performance of this model for AD stage classification was insufficient; therefore, we modified it with the latest concept named Self-Attention.

**Figure 8:**
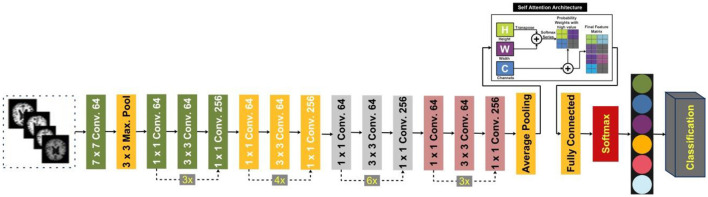
Proposed ResNet-Self architecture for classification of AD stages.

The proposed ResNet-Self architecture is illustrated in Figure 8. This figure shows that the self-attention layer was added after the global average pooling layer. A flattening layer has been added before the self-attention layer that converts the input into 1D. The first channel is passed to the Softmax function that combines with the second channel for the attention map creation. After that, the generated attention map is combined with a third channel for final attention features that are further utilized to classify AD stages.

##### 3.2.5.1 Self-attention

The internal attention approach, sometimes called the self-attention (SA) strategy, uses internal information to automatically identify and highlight relevant information without needing external information. SA has low computational complexity and allows parallel computing. It consists of three characteristics matrices such as *X, Y*, and *V*, where these are defined by Equations 5–9.


(5) $$\begin{array}{l} \left\{Y,X\right\}\in R^{T\times T} \end{array}$$

(6) $$\begin{array}{l} V\in R^{T\times J} \end{array}$$

Initially, the correlation score has been computed among all rows of Y and X as follows:


(7) $$\begin{array}{l} P=XY^{\tau } \end{array}$$

where *Y*^τ^ denotes the transpose of *Y* and *P*∈*R*^*T*×*T*^. The softmax function is applied in the next step, which converts the correlation score into probability values. Mathematically, it is formulated as follows:


(8) $$\begin{array}{l} SM\left(P\right)\left(i,j\right)=\frac{e^{P\left(i,j\right)}}{\overset{T-1}{\underset{j=0}{\sum }}e^{P\left(i,j\right)}} \end{array}$$

Hence, the final attention map has been obtained as follows:


(9) $$\begin{array}{l} AMp=SM\left(P\right)V \end{array}$$

##### 3.2.5.2 Proposed network training

After the design of the proposed model, the next step is training a model using the deep transfer learning concept. The entire model is trained from scratch, instead of any frozen layer. The hyperparameters of this network are presented in Table 3. Based on the selected hyperparameters using BO, the proposed model is trained on the augmented dataset. The best-returned value of the learning rate using BO is 0.00032, and the momentum value is 0.773. After the training process, the test data are employed for the extraction of the features.

##### 3.2.5.3 Deep features extraction

Typically, CNNs return the three levels of feature maps: low-level feature maps, mid-level feature maps, and high-level feature maps. All of these levels contain different information. Low-level feature maps contain simple patterns such as edges, corners, and textures. These maps have high dimensions. Mid-level feature maps contain abstract and more structured patterns like specific regions of the objects or textures, but high-level features contain discriminative and semantically significant information. The features (high-level) are extracted from the last stage of CNNs due to their lower dimensions. The low dimensionality reduces the memory requirement and computational complexity.

This study extracts deep features using the self-attention layer, instead of the global average pooling layer. The self-attention layer returns the prominent and relevant global information within the images. The testing images are utilized, and a trained model is opted for. The batch size was 128 during the deep features extraction. The self-attention layer features contain deeper information about the AD stages. The size of the extracted feature vector is *N* × 2,048. The extracted features are analyzed and optimized using an improved pathfinder optimization algorithm.

#### 3.2.6 Best features selection

In this study, we utilized an evolutionary optimization algorithm named Entropy Path Finder Optimization (EPFO) for the best feature selection. Features are selected at the initial step through original pathfinder optimization and later refined using an entropy approach that handles the uncertainty.

##### 3.2.6.1 Path finder optimization

In contrast to previously suggested swarm intelligence, the Pathfinder approach does not specify which species group it belongs to. For instance, the seagull optimization algorithm restricts the number of seagulls, whereas the gray wolf optimization technique restricts the number of gray wolves, etc. The Pathfinder algorithm is based on many surviving traits and regulations of animals. Based on the best fitness of the organism, the Pathfinder algorithm divides cluster animals among two sorts of tasks: the leader (only with the lowest fitness value) and the follower. The leader must find the greatest food and label it for the followers. The markings left behind by the Pathfinder are used as a reference point by the followers, who then proceed to follow the Pathfinder. Hence, both the Pathfinder and the follower are skeptical. That is why the two distinct sorts of responsibilities may switch places with one another depending on the individual's level of search capability as the number of iterative steps of the method rises; that is, those who lead the way sometimes get followers. Similarly, followers may also play the role of a pathfinder. To optimize a task, the PFA is split into two segments. The initial stage is a period of exploration. The PFA changes the location using the following Equation (10):


(10) $$\begin{array}{l} x_{p}^{k+1}\;=x_{p}^{k}\;+\;2r3\;.\;\left(x_{p}^{k}-\;x_{p}^{k-1}\right)\;+A \end{array}$$

where $x_{p}^{k+1}$ demonstrates the modified position vector of PFA. $x_{p}^{k}$ indicates the present location of vectors in PFA, while $x_{p}^{k-1}$ shows the former position of the vector of PFA. The ongoing iteration count is denoted by the variable *k*. *R*3 is a random vector that is created in a uniform manner in the range [0,1], whereas A is produced within every iteration by applying Equation (11). Step 2 is really the exploitation step, which is immediately preceded by the location change. The following update formula applies Equation (11):


(11) $$\begin{array}{l} x_{i}^{k+1}=\;x_{i}^{k}\;+R1\;.\;\left(x_{j}^{k}\;+\;x_{i}^{k}\right)+R2.\;\left(x_{p}^{k}\;+\;x_{i}^{k}\right)\\ \;+\;E,\;i\;\geq \;2 \end{array}$$

where $x_{i}^{k+1}$ indicates the updated location vector of the i-th integer just after location modification. $x_{i}^{k}$ is the location vector of the i-th individual, $x_{j}^{k}$ is the neighboring individual, and $x_{p}^{k}$ is the Pathfinder. The variable k denotes the ongoing iteration count. Each of the vectors R1 and R2 is completely unpredictable. In this situation, *R*_1_ = (α*r*1) and *R*_2_= (β*r*2), and here, *R*_1_ and *R*_2_ are random vectors that are created uniformly in the range [0,1]. α determines the degree to which each component travels about its neighbors and is hence called the coefficient of iteration. β establishes a randomized spacing to make the herd fairly constant along with the leader and hence called the coefficient of attraction k_max_. Mathematically, it is formulated by Equation (12).


(12) $$\begin{array}{l} h\;=\left(1-\frac{k}{kmax}\right)\;.\;\mu_{1}\;.\;D_{ij},\;D_{ij\;}||x_{i}\;-\;x_{j}|| \end{array}$$

Therefore, here μ_1_ and μ_2_ are randomly generated two vectors in the interval of [1,1], *D*_*ij*_ is the gap between both individuals, (*k*) denotes the present iteration range, and k_max_ is the maximal quantity of repetitions. (*A*) and ( *h* ) may give random walk strides for all persons when the second part of Equations (10) and (11) and the third part of Equation (12) are equal to zero. As a result, in order to ensure that the motion will be in several directions and completely random, the values of. (*A*) and (*h* ) should be within the proper span.

After every update in the position, the KNN classifier is employed to measure the fitness value. The cost function of KNN is mathematically formulated as:


(13) $$\begin{array}{l} \tau_{cost}=\varphi_{\alpha }\times \epsilon_{err}+\varphi_{\beta }\times \left(\frac{count\;of\;sel\text{\_}feat}{Max\left(features\right)}\right) \end{array}$$

where α and β are denoted, the coefficient having values are 0.94 and 0.014, respectively. The ϵ_*err*_ presented the error value that is calculated by employing an Equation (14):


(14) $$\begin{array}{l} \epsilon_{err}=\;1-\partial_{accuracy} \end{array}$$

##### 3.2.6.2 Entropy selection

Assume *U* is a discrete random variable, and it is represented as *u* = {*u*_1_, *u*_2_, …, *u*_*n*_}, then if an element *u*_*i*_ occurs with *p*(*u*_*i*_), the entropy *H*(*U*) of *U* is formulated by Equation (15):


(15) $$\begin{array}{l} H\left(U\right)=-\overset{n}{\underset{i=1}{\sum }}p\left(u_{i}\right)\log p\left(u_{i}\right) \end{array}$$

where *n* denotes the total number of features. The fuzzy C-Means clustering is utilized to construct the membership function of all features. The fuzzy membership method is defined in the following five steps.

In the first step, we assumed the number of clusters (C), where 2 ≤ *C* ≤ *N*. In the next step, the *jth* center clusters are computed by the following Equation (16).


(16) $$\begin{array}{l} C_{j}=\frac{\overset{N}{\underset{i=1}{\sum }}\mu_{ij}^{e}u_{ij}}{\overset{N}{\underset{i=1}{\sum }}\mu_{ij}^{g}} \end{array}$$

where *e*≥1 is a fuzziness coefficient and μ_*ij*_ is the degree of membership (DOM) for the *ith* data point *u*_*i*_ in *jth* cluster. Euclidean distance is computed in the third step using Equation (17).


(17) $$\begin{array}{l} D_{ij}=|C_{j}-\mu_{i}| \end{array}$$

In the fourth step, the value of the fuzzy membership function is updated by Equation (18):


(18) $$\begin{array}{l} \mu =\frac{1}{\overset{e}{\underset{m=1}{\sum }}{\left(\frac{D_{ij}}{D_{im}}\right)}^{\frac{2}{g-1}}} \end{array}$$

In the final step, we repeated steps 2–4 until the change in μ was less as per the previous values. Hence, the fuzzy entropy function is formulated as follows in Equation (19):


(19) $$Fe\left(\overset{\vee }{H}\right)=-\lambda_{c}\left(\overset{\vee }{H}\right)\log\lambda_{c}\left(\overset{\vee }{H}\right)$$

where $\lambda_{c}\left(\overset{Ȟ}{\;}\right)$ is a class degree of the membership function (Khushaba et al., 2007). The fuzzy entropy process is applied to the selected features of Equation (12). The dimensions of the selected features are *N* × 1,467. The final features are employed for the classification. The proposed fuzzy entropy-controlled pathfinder algorithm's pseudo-code is given in Algorithm 1.

**Algorithm 1:**
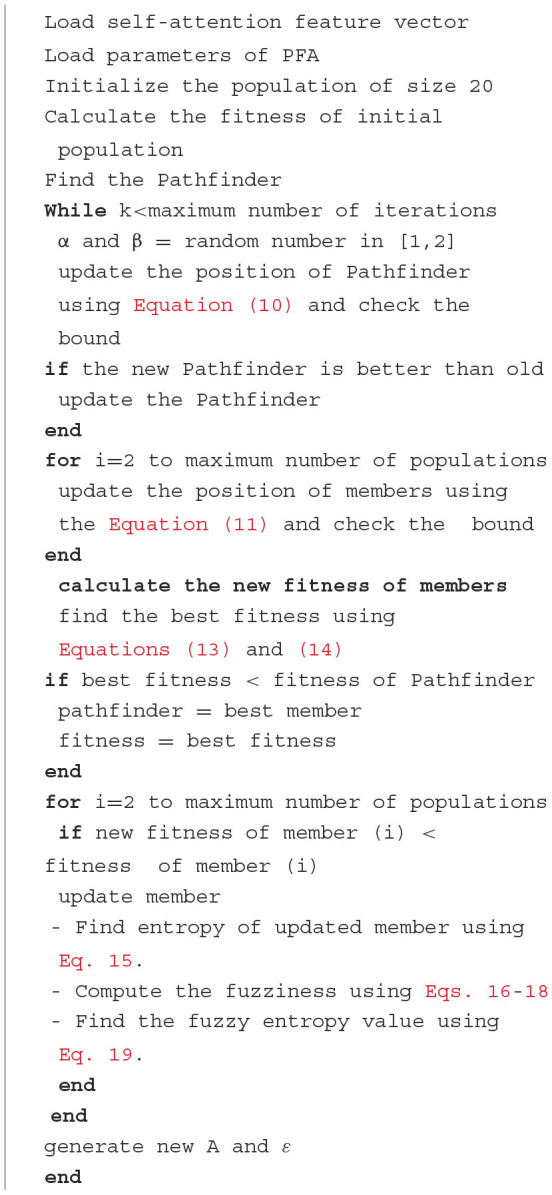
The pathfinder algorithm.

## 4 Results and analysis

The proposed AD stage classification model undergoes evaluation using a Kaggle dataset, providing a robust framework for assessing its performance. The forthcoming section will comprehensively showcase all the experiments conducted and the corresponding results obtained, offering insights into the efficacy and potential of the proposed model in accurately diagnosing AD.

### 4.1 Experimental setup and evaluation measures

The experimental process of this study is discussed here. The proposed framework of AD is evaluated on a publically available dataset that includes four classes as mentioned in Section 3.1. The dataset is divided into 50:50 approaches, and training data augmentation is performed. The training data extracts and optimizes features for the best feature selection. The selected features are classified using machine learning classifiers, and the following measures are computed: recall rate, precision rate, F1-Score, MCC, and KAPPA. The entire experimental process has been conducted on MATLAB2023a using a personal computer with 128GB RAM, 512FB SSD, and a 12GB Graphics Card of NVIDIA3060 RTX.

### 4.2 Proposed ResNet-Self results (random values)

The proposed ResNet-Self CNN architecture is tested on 1,600 images in this experiment. The hyperparameters of this experiment are randomly initialized (related work knowledge such as learning rate 0.0001 and momentum 0.70) and performed training. Features are extracted from the testing data, and the maximum accuracy of 99.93% for the NNN classifier was obtained (results seen in Table 4). The values of precision measure are 99.93%, and the Kappa value is 99.80%, respectively. The computational time taken by the NNN classifier is 33.532 (s), whereas the minimum noted time is 28.071 (s) for bilayered NN. The rest of the classifiers obtained accuracies of 98.7, 99.90, 99.65, 98.97, 73.86, 98.75, 99.88, and 99.72%, respectively.

**Table 4:** Proposed prediction results of AD stages using initialization of random hyperparameters.

**S#**	**Classifiers**	**Precision**	**Recall**	**F1-score**	**Kappa**	**MCC**	**Accuracy**	**Time (s)**
1	Fine KNN	98.73	98.73	98.72	96.60	98.30	98.7	66.002
2	**NNN**	**99.93**	**99.93**	**99.92**	**99.80**	**99.90**	**99.93**	**33.532**
3	MNN	99.90	99.90	99.90	99.73	99.87	99.90	50.399
4	Trilayered NN	99.65	99.65	99.65	99.07	99.53	99.65	41.09
5	Medium KNN	98.99	98.97	98.97	97.27	98.64	98.97	62.531
6	Coarse KNN	83.00	97.60	87.50	30.31	84.03	73.86	63.041
7	Cosine KNN	98.78	98.75	98.75	96.67	98.35	98.75	66.134
8	Bilayered NN	99.88	99.88	99.87	99.67	99.83	99.88	28.071
9	Medium Gaussian SVM	99.73	99.72	99.73	99.27	99.63	99.72	73.511

Bold values shows the best results.

### 4.3 Bayesian optimization results

This section presents the results obtained from Bayesian optimization (BO). We executed our BO algorithm 100 times and got the value for a learning rate of 0.00010195, momentum value of 0.81079, L2Regularization of 2.8724e^−10^, and section depth value of 3. These are the best feasible points. Based on these points, the classification was performed, and the results are noted in Table 5. The MNN classifier achieved a maximum accuracy of 99.95% in this table. The precision rate of this classifier is 99.95, the Kappa value of 99.87, and the MCC value of 99.95%, respectively. In addition, the computation time of this classifier is 17.78 (s). Compared to the results in Table 4, this experiment shows improved accuracy, precision, Kappa, and MCC values. Moreover, the computation time of this experiment was less than that of the results in Table 4. The results show that selecting hyperparameters using BO can improve the accuracy and reduce the computational cost.

**Table 5:** Proposed classification results after employing Bayesian optimization-based selection of hyperparameters.

**S#**	**Classifiers**	**Precision**	**Recall**	**F1-score**	**Kappa**	**MCC**	**Accuracy**	**Time**
1	Fine KNN	98.48	98.48	98.47	95.93	97.97	98.48	35.573
2	NNN	99.90	99.90	99.90	99.73	99.87	99.90	15.874
3	**MNN**	**99.95**	**99.95**	**99.95**	**99.87**	**99.93**	**99.95**	**17.78**
4	Trilayered NN	99.75	99.75	99.75	99.33	99.67	99.75	21.891
5	Medium KNN	98.75	98.72	98.72	96.60	98.31	98.72	34.139
6	Coarse KNN	97.11	97.08	97.07	92.20	96.11	97.08	34.571
7	Cosine KNN	98.45	98.40	98.40	95.73	97.89	98.40	37.183
8	Bilayered KNN	99.58	99.58	99.58	98.87	99.43	99.58	34.292
9	Medium Gaussian SVM	99.73	99.72	99.73	99.27	99.63	99.72	34.235

Bold values shows the best results.

### 4.4 Proposed feature selection

Table 6 presents the AD stage classification results using the proposed selection of BO extracted features. In the first stage of this table, results are presented for the original pathfinder algorithm. The PFA was applied to the BO-based deep features extraction and performed classification. The maximum obtained accuracy for this experiment is 99.82%. The precision and recall values are 99.83 and 99.83%. In addition, Kappa and MCC measure values of 99.80 and 99.80%, respectively. Compared to Tables 4, 5, the selection results show better. Moreover, the computation time of each classifier is also noted, and the minimum noted time for this experiment is 12.338 (s), which is less than Tables 4, 5. Overall, the time is decreased after employing the optimization method.

**Table 6:** Proposed classification results after employing Bayesian optimization and proposed feature selection algorithm.

**S#**	**Classifiers**	**Precision**	**Recall**	**F1-score**	**Kappa**	**MCC**	**Accuracy**	**Time**
**Features selection using original PFA**								
1	Fine KNN	94.34	94.32	94.32	84.82	92.43	94.31	23.406
2	**NNN**	**99.83**	**99.83**	**99.82**	**99.80**	**99.80**	**99.82**	**12.338**
3	MNN	99.82	99.80	99.82	99.47	99.80	99.80	14.71
4	Trilayered NN	99.80	99.80	99.80	99.47	99.73	99.80	16.088
5	Medium KNN	98.94	98.92	98.92	97.13	98.57	98.92	14.9
6	Coarse KNN	96.64	96.57	96.57	90.87	95.46	96.57	14.67
7	Cosine KNN	98.73	98.70	98.70	96.53	98.28	98.70	19.92
8	Bilayered KNN	99.88	99.88	99.87	99.67	99.83	99.80	15.04
9	Medium Gaussian SVM	99.73	99.73	99.73	99.27	99.63	99.73	17.014
**Features selection using proposed fuzzy entropy PFA**								
1	Fine KNN	99.90	99.90	99.90	99.73	99.87	99.90	18.987
2	**NNN**	**99.93**	**99.92**	**99.92**	**99.80**	**99.90**	**99.90**	**10.231**
3	MNN	99.90	99.90	99.90	99.73	99.87	99.90	13.395
4	Trilayered NN	99.73	99.73	99.72	99.27	99.63	99.73	11.411
5	Medium KNN	99.04	99.03	99.03	97.41	98.71	99.03	7.167
6	Coarse KNN	97.05	97.00	96.99	92.00	96.02	97.00	6.77
7	Cosine KNN	98.74	98.70	98.70	96.53	98.28	98.70	8.683
8	Bilayered KNN	99.85	99.82	99.85	99.60	99.80	99.85	10.336
9	Medium Gaussian SVM	99.70	99.70	99.70	99.20	99.60	99.70	9.118

Bold values shows the best results.

**Figure 9:**
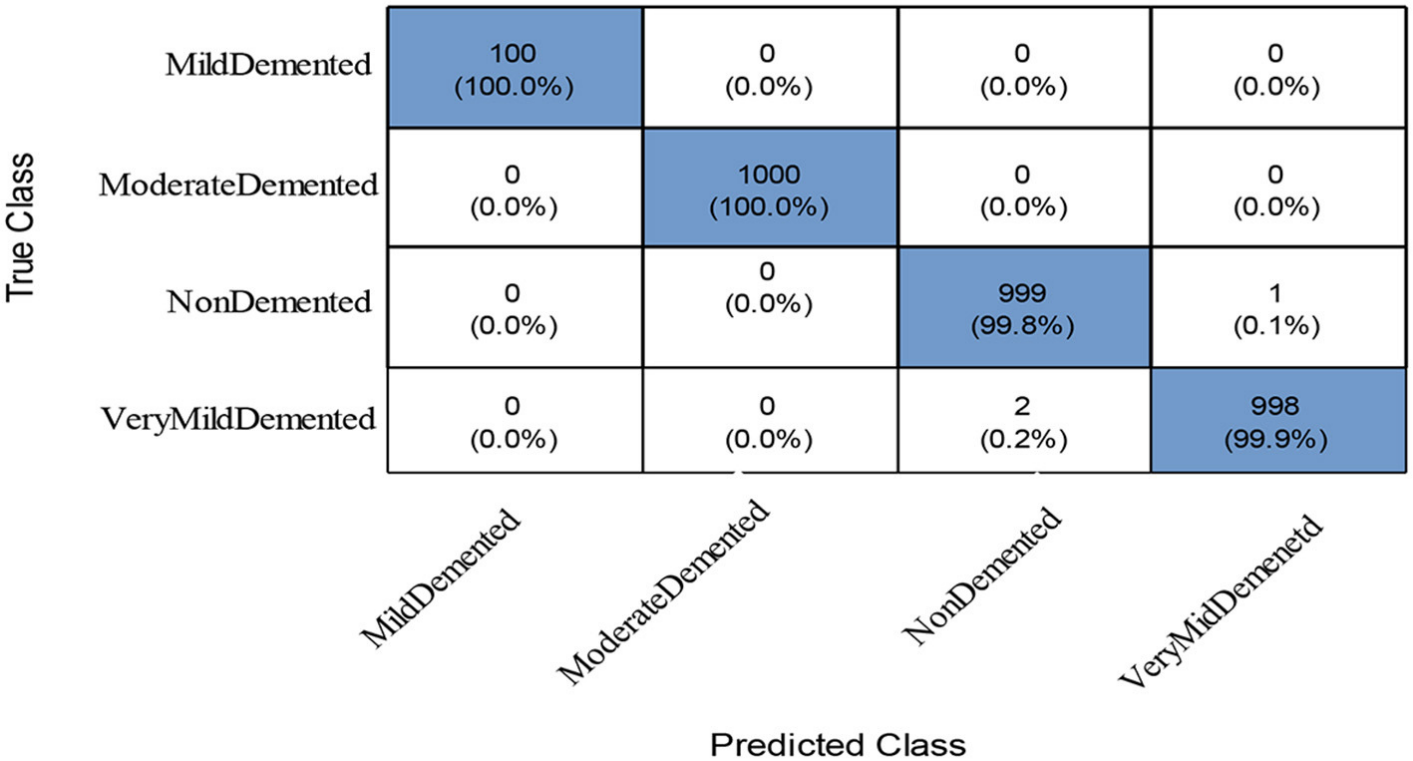
Confusion matrix of the NNN classifier using proposed feature selection algorithm.

To further improve (minimize) the computational time, we improved the PFA using Fuzzy Entropy formulation in this study. The proposed Fuzzy Entropy PFA (FEPFA) results are given in the second half of Table 6. The maximum obtained accuracy for this technique is 99.90%, whereas the precision rate value of 99.93%. The Kappa and MCC values of this experiment are 99.80 and 99.90%, respectively. In addition, the computation time of this classifier is 10.231 (s), less than the original PFA (12.338). Overall, the performance of this technique is improved and time is minimized. The performance of the NNN classifier can be further verified using a confusion matrix illustrated in Figure 9. In this figure, the diagonal values represent the true predicted rates of each class.

### 4.5 Discussion and comparison

**Figure 10:**
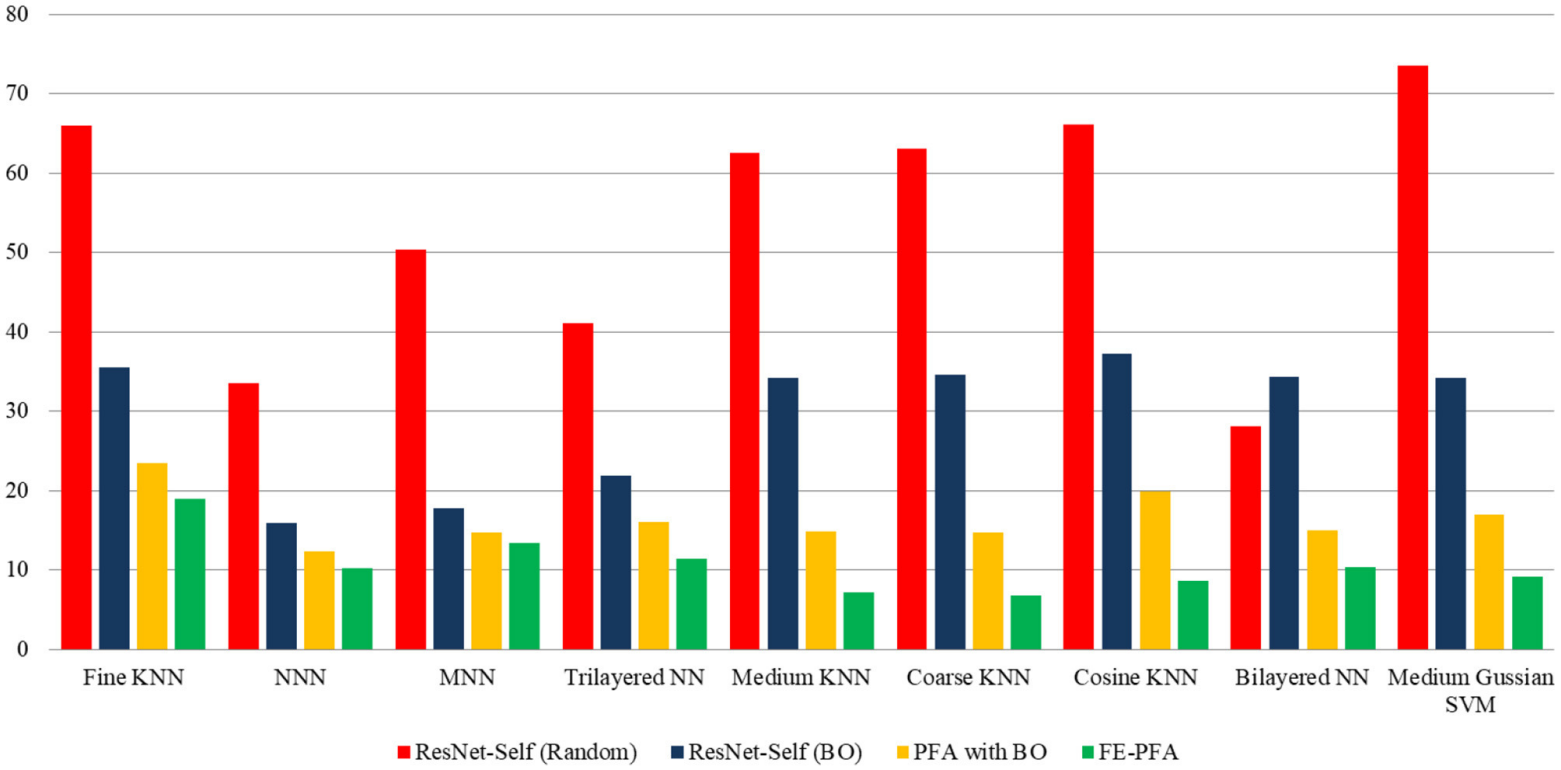
Comparison among middle steps of the proposed method in terms of computational time.

In this section, a detailed analysis of the proposed study has been conducted in the form of visual graphs and comparison with recent state-of-the-art (SOTA) techniques. The proposed framework of AD stage classification has been discussed in Section 3.1, and the visual illustration is shown in Figure 1. The MRI dataset has been used for the experimental process (a few sample images are shown in Figures 2, 3). The augmentation process has been performed to increase the number of images for a better training process. After that, a new model is proposed named ResNet-Self as shown in Figure 8 for the accurate classification of AD stages. The performance of AD stage classification is improved by proposing new FEPFA techniques that select the best features. The results are presented in Tables 4–6. Table 4 presents results for the proposed ResNet-Self architecture using random initialization of hyperparameters. Table 5 presents the results of the proposed ResNet-Self after employing BO for hyperparameters selection. Table 5 shows better accuracy, precision rate, MCC, and Kappa performance than Table 4. The computational time and precision rate are further improved using the proposed FEPFA feature selection algorithm, and the results are presented in Table 6. In this table, accuracy is also improved and time is significantly decreased. In addition, a comparison is also conducted of the proposed FEPFA with the original PFA, showing the improvement in accuracy, precision, MCC, and computational time. Overall, the time comparison is illustrated in Figure 10. This figure clearly shows that the proposed selection method consumed less time than the other steps.

Table 7 compares the methods currently utilized for predicting AD. To enhance the categorization of early AD phases while reducing parameters and computational costs, a novel detection network named DAD-Net was introduced by Mohi et al. (2023). This network appropriately classified initial AD processes and depicted class activation characteristics as a heat map of the brain, achieving 99.2% accuracy using a Kaggle dataset. Additionally, AI-Atroshi et al. (2022) utilized convolutional layers with freeze elements from ImageNet, achieving 99.27% accuracy on ADNI's MRI data collection for both binary and ternary classification. Authors in Shankar et al. (2022) employed a ResNet-18 architecture using a transfer learning concept and obtained an accuracy of 83.3% on Kaggle datasets. Authors in Sharma et al. (2022) utilized a CNN-based pre-trained network named ResNet-50 and achieved 91.78% accuracy. Authors in Albright (2019) proposed a ResNet-15 model and fused it with DenseNet-169 for the classification of AD prediction. They achieved an improved accuracy of 88.70% on Kaggle's AD dataset. Furthermore, Soliman et al. (2022) suggested a novel approach employing three pre-trained CNN frameworks such as DenseNet196, VGG16, and ResNet-50, achieving 89% accuracy on MRI brain data from Kaggle. Hashmi (2024) proposed a compact architecture by merging LeNet and AlexNet models, achieving 93.58% accuracy on the ADNI dataset. Goel et al. (2023) proposed a system for automated AD diagnosis, integrating multiple customized deep-learning models. This architecture achieved 96.61% accuracy using rs-fMRI datasets and modified AlexNet and Inception blocks. Ismail et al. (2023) utilized a new optimized ensemble-based DNN learning model named MultiAz-Net and obtained 92.3% accuracy on the ADNI dataset.

**Table 7:** Comparison of proposed method results with existing techniques.

**References**	**Years**	**Models**	**Datasets**	**Results**
Ahmed et al. (2022)	2022	CNN based DAD-Net	Kaggle	99.22%
Naz et al. (2022)	2022	CNN using freeze features	ADNI	99.27%
Oktavian et al. (2002)	2022	CNN with ResNet-18	Kaggle	83.3%
Ebrahimi et al. (2021)	2021	CNN, ResNet-18, temporalCN, RNN	ImageNet	91.78%
Al Shehri (2022)	2022	ResNet-15	Kaggle	88.70%
Techa et al. (2022)	2022	ResNet-15	Kaggle	89%
Abunadi (2022)	2022	ResNet-18, AlexNet	Kaggle	99.94%
Prasath and Sumathi (2024)	2023	LeNet, AlexNet	ADNI	93.58%
Sorour et al. (2024)	2023	AlexNet, Inception blocks	ADNI	96.61%
Ismail et al. (2023)	2023	MOGOA	ADNI	92.3%
**Proposed model**		**ResNet-50**	**Kaggle**	**99.99%**

Bold values shows the best results.

## 5 Conclusion and future study

It is challenging to diagnose and predict Alzheimer's disease using multiclass datasets promptly. A computerized technique is widely required for early AD prediction from MRI images. This study proposes a computerized framework based on deep-learning and optimization algorithms. A dataset balancing issue has been resolved at the initial stage using mathematical formulations that improved the training capability of the proposed ResNet-Self deep model. The proposed ResNet-Self model is a combination of ResNet-50 architecture modified by adding the self-attention module. The self-attention module shows improved accuracy; however, the random initialization of hyperparameters impacts the accuracy and computational time. Therefore, we implemented a BO technique that automatically initialized the hyperparameters for the training process. Moreover, we proposed a feature selection algorithm named FEcPFA that selects the best features and shows improved accuracy (99.90), precision rate, and Kappa value. In addition, the computational time is significantly reduced, which is the strength of FEcPFA. The optimized hyperparameters that make the proposed model less generalized and lead to overfitting are the limitations of the proposed framework. In the future, a new custom model will be proposed based on the fire module, and the output of that module will be employed with self-attention and cross-validation to overcome overfitting. In addition, more MRI datasets will be utilized for the experimental process.

## Data availability statement

The original contributions presented in the study are included in the article/supplementary material, further inquiries can be directed to the corresponding authors.

## Author contributions

NY: Methodology, Project administration, Resources, Software, Writing—original draft. MK: Methodology, Project administration, Resources, Software, Validation, Writing—original draft. SM: Formal analysis, Investigation, Methodology, Resources, Software, Writing—original draft. HA: Conceptualization, Data curation, Methodology, Project administration, Software, Writing—review & editing. AH: Data curation, Methodology, Software, Validation, Writing—original draft. FA: Conceptualization, Data curation, Funding acquisition, Investigation, Methodology, Visualization, Writing—review & editing. LJ: Conceptualization, Funding acquisition, Methodology, Software, Supervision, Validation, Visualization, Writing—review & editing. AM: Funding acquisition, Methodology, Software, Validation, Writing—original draft.

## References

[B1] Abunadi I. (2022). Deep and hybrid learning of MRI diagnosis for early detection of the progression stages in Alzheimer's disease. Conn. Sci. 34, 2395–2430. 10.1080/09540091.2022.212345037175045

[B2] Ahmed G. Er M. J. Fareed M. M. S. Zikria S. Mahmood S. He J.. (2022). Dad-net: classification of alzheimer's disease using adasyn oversampling technique and optimized neural network. Molecules 27:7085. 10.3390/molecules27207085 36296677 PMC9611525

[B3] AI-Atroshi C. Rene Beulah J. Singamaneni K. K. Pretty Diana Cyril C. Neelakandan S. Velmurugan S. (2022). Automated speech based evaluation of mild cognitive impairment and Alzheimer's disease detection using with deep belief network model. Int. J. Healthc. Manag. 21, 1–11. 10.1080/20479700.2022.2097764

[B4] Al Shehri W. (2022). Alzheimer's disease diagnosis and classification using deep learning techniques. PeerJ Comp. Sci. 8:e1177. 10.7717/peerj-cs.1177 37346304 PMC10280208

[B5] Albright J. (2019). Forecasting the progression of Alzheimer's disease using neural networks and a novel pre-processing algorithm. Alzheimers Dement 5, 483–491. 10.1016/j.trci.2019.07.001 31650004 PMC6804703

[B6] Balaji P. Chaurasia M. A. Bilfaqih S. M. Muniasamy A. Alsid L. E. G. (2023). Hybridized deep learning approach for detecting Alzheimer's disease. Biomedicines 11:149. 10.3390/biomedicines11010149 36672656 PMC9855764

[B7] Bloniecki Kallio V. (2002). Using CSF Biomarkers to Understand Mechanisms of behAvioral Changes and Effects of Drug Treatment in Dementia.

[B8] Bondi M. W. Edmonds E. C. Salmon D. P. (2017). Alzheimer's disease: past, present, and future. J. Int. Neuropsychol. Soc. 23, 818–831. 10.1017/S135561771700100X 29198280 PMC5830188

[B9] Carle P. (2022). Senescent Human Astrocytes Produce Amyloid-Beta in a G3BP1-Dependent Manner. McGill University.

[B10] Chua J. J. E. (2023). HEBP1-An early trigger for neuronal cell death and circuit dysfunction in Alzheimer's disease. Semin. Cell Dev. Biol. 139, 102–110. 10.1016/j.semcdb.2022.07.005 35842370

[B11] Dhakhinamoorthy C. Mani S. K. Mathivanan S. K. Mohan S. Jayagopal P. Mallik S.. (2023). Hybrid whale and gray wolf deep learning optimization algorithm for prediction of Alzheimer's disease. Mathematics 11:1136. 10.3390/math11051136

[B12] Ebrahimi A. Luo S. Chiong R. (2021). Deep sequence modelling for Alzheimer's disease detection using MRI. Comput. Biol. Med. 134:104537. 10.1016/j.compbiomed.2021.104537 34118752

[B13] Fabrizio C. Termine A. Caltagirone C. Sancesario G. (2021). Artificial intelligence for Alzheimer's disease: promise or challenge?. Diagnostics 11:1473. 10.3390/diagnostics11081473 34441407 PMC8391160

[B14] Ghazal T. M. Abbas S. Munir S. Khan M. A. Ahmad M. Issa G. F.. (2022). Alzheimer disease detection empowered with transfer learning. Comp. Mater. Continua 70:020866. 10.32604/cmc.2022.02086637303558

[B15] Goel T. Sharma R. Tanveer M. Suganthan K. Pilli R. (2023). Multimodal neuroimaging based Alzheimer's disease diagnosis using evolutionary RVFL classifier. IEEE J. Biomed. Health Inf. 3, 1–21. 10.1109/JBHI.2023.3242354 37022418

[B16] Gómez-Isla T. Frosch M. P. (2022). Lesions without symptoms: understanding resilience to Alzheimer disease neuropathological changes. Nat. Rev. Neurol. 18, 323–332. 10.1038/s41582-022-00642-9 35332316 PMC10607925

[B17] Hashmi S. A. (2024). Malware detection and classification on different dataset by hybridization of CNN and machine learning. Int. J. Intell. Syst. Appl. Eng. 12, 650–667. Available online at: https://ijisae.org/index.php/IJISAE/article/view/400436262124

[B18] Hazarika R. A. Maji A. Kandar D. Jasińska E. Krejci P. Leonowicz Z.. (2023). An approach for classification of Alzheimer's disease using deep neural network and brain magnetic resonance imaging (MRI). Electronics 12:676. 10.3390/electronics12030676

[B19] Hoozemans J. Veerhuis R. Rozemuller J. Eikelenboom P. (2006). Neuroinflammation and regeneration in the early stages of Alzheimer's disease pathology. Int. J. Dev. Neurosci. 24, 157–165. 10.1016/j.ijdevneu.2005.11.001 16384684

[B20] Ismail W. N. Rajeena P. P. F. Ali M. A. (2023). A meta-heuristic multi-objective optimization method for Alzheimer's disease detection based on multimodal Data. Mathematics 11:957. 10.3390/math11040957

[B21] Jansi R. Gowtham N. Ramachandran S. Praneeth V. S. (2023). “Revolutionizing Alzheimer's disease prediction using InceptionV3 in deep learnin,” in 2023 7th International Conference on Electronics, Communication and Aerospace Technology (ICECA) (IEEE), 1155–1160.

[B22] Jia H. Lao H. (2022). Deep learning and multimodal feature fusion for the aided diagnosis of Alzheimer's disease. Neur. Comp. Appl. 34, 19585–19598. 10.1007/s00521-022-07501-0

[B23] Jo T. Nho K. Bice P. Saykin A. J. Alzheimer's Disease Neuroimaging Initiative (2022). Deep learning-based identification of genetic variants: application to Alzheimer's disease classification. Brief. Bioinform. 23:bbac022. 10.1093/bib/bbac022 35183061 PMC8921609

[B24] Kasula B. Y. (2023). A machine learning approach for differential diagnosis and prognostic prediction in Alzheimer's disease. Int. J. Sustain. Dev. Comp. Sci. 5, 1–8. Available online at: https://www.ijsdcs.com/index.php/ijsdcs/article/view/397

[B25] Kellar D. Craft S. (2020). Brain insulin resistance in Alzheimer's disease and related disorders: mechanisms and therapeutic approaches. Lancet Neurol. 19, 758–766. 10.1016/S1474-4422(20)30231-3 32730766 PMC9661919

[B26] Khalid A. Senan E. M. Al-Wagih K. Ali Al-Azzam M. M. Alkhraisha Z. M. (2023). Automatic analysis of MRI images for early prediction of Alzheimer's disease stages based on hybrid features of CNN and handcrafted features. Diagnostics 13:1654. 10.3390/diagnostics13091654 37175045 PMC10178535

[B27] Khushaba R. N. Al-Jumaily A. Al-Ani A. (2007). “Novel feature extraction method based on fuzzy entropy and wavelet packet transform for myoelectric control,” in 2007 International Symposium on Communications and Information Technologies (IEEE), 352–357.

[B28] Koul A. Bawa R. K. Kumar Y. (2023). An analysis of deep transfer learning-based approaches for prediction and prognosis of multiple respiratory diseases using pulmonary images. Arch. Comp. Methods Eng. 6, 1–27. 10.1007/s11831-023-10006-1 PMC10249943 37359745

[B29] Mahmud T. Barua K. Habiba S. U. Sharmen N. Hossain M. S. Andersson K.. (2024). An explainable AI paradigm for Alzheimer's diagnosis using deep transfer learning. Diagnostics 14:345. 10.3390/diagnostics14030345 38337861 PMC10855149

[B30] Marwa E.-G. Moustafa H. E.-D Khalifa F. Khater H. (2023). An MRI-based deep learning approach for accurate detection of Alzheimer's disease. Alexandria Eng. J. 63, 211–221. 10.1016/j.aej.2022.07.062

[B31] Mirzaei G. Adeli H. (2022). Machine learning techniques for diagnosis of alzheimer disease, mild cognitive disorder, and other types of dementia. Biomed. Signal Process. Control 72:103293. 10.1016/j.bspc.2021.103293

[B32] Mohammad F. Al Ahmadi S. (2023). Alzheimer's disease prediction using deep feature extraction and optimisation. Mathematics 11:3712. 10.3390/math11173712

[B33] Mohi G. Bhagat A. Ansarullah S. I. Othman M. T. B. Hamid Y. Alkahtani H. K.. (2023). A novel framework for classification of different Alzheimer's disease stages using CNN model. Electronics 12:469. 10.3390/electronics12020469

[B34] Nagdee M. (2011). Dementia in intellectual disability: a review of diagnostic challenges. Afr. J. Psychiatry 14, 194–199. 10.4314/ajpsy.v14i3.1 21863203

[B35] Naz S. Ashraf A. Zaib A. (2022). Transfer learning using freeze features for Alzheimer neurological disorder detection using ADNI dataset. Multim. Syst. 28, 85–94. 10.1007/s00530-021-00797-3

[B36] Oktavian M. W. Yudistira N. Ridok A. (2002). Classification of Alzheimer's disease using the convolutional neural network (CNN) with transfer learning and weighted loss. arXiv [preprint]. 10.48550/arXiv.2207.01584

[B37] Perumal S. Velmurugan T. (2018). Pre-processing by contrast enhancement techniques for medical images. Int. J. Pure Appl. Math. 118, 3681–3688. Available online at: https://www.researchgate.net/profile/Velmurugan-Thambusamy/publication/325361080_Preprocessing_by_contrast_enhancement_techniques_for_medical_images/links/5fc1ee66a6fdcc6cc6774288/Preprocessing-by-contrast-enhancement-techniques-for-medical-images.pdf

[B38] Prasath T. Sumathi V. (2024). Pipelined deep learning architecture for the detection of Alzheimer's disease. Biomed. Signal Process. Control 87:105442. 10.1016/j.bspc.2023.105442

[B39] Rathore S. Habes M. Iftikhar M. A. Shacklett A. Davatzikos C. (2017). A review on neuroimaging-based classification studies and associated feature extraction methods for Alzheimer's disease and its prodromal stages. Neuroimage 155, 530–548. 10.1016/j.neuroimage.2017.03.057 28414186 PMC5511557

[B40] Samhan L. F. Alfarra A. H. Abu-Naser S. S. (2022). Classification of Alzheimer's Disease Using Convolutional Neural Networks.

[B41] Shamrat F. M. J. M. Akter S. Azam S. Karim A. Ghosh P. Tasnim Z.. (2023). AlzheimerNet: An effective deep learning based proposition for alzheimer's disease stages classification from functional brain changes in magnetic resonance images. IEEE Access 11, 16376–16395. 10.1109/ACCESS.2023.3244952

[B42] Shankar V. G. Sisodia D. S. Chandrakar P. (2022). A novel discriminant feature selection–based mutual information extraction from MR brain images for Alzheimer's stages detection and prediction. Int. J. Imaging Syst. Technol. 32, 1172–1191. 10.1002/ima.22685

[B43] Shanmugam J. V. Duraisamy B. Simon B. C. Bhaskaran P. (2022). Alzheimer's disease classification using pre-trained deep networks. Biomed. Signal Process. Control 71:103217. 10.1016/j.bspc.2021.103217

[B44] Sharma R. Goel T. Tanveer M. Murugan R. (2022). FDN-ADNet: Fuzzy LS-TWSVM based deep learning network for prognosis of the Alzheimer's disease using the sagittal plane of MRI scans. Appl. Soft Comput. 115:108099. 10.1016/j.asoc.2021.108099

[B45] Shaukat N. Amin J. Sharif M. Azam F. Kadry S. Krishnamoorthy S.. (2022). Three-dimensional semantic segmentation of diabetic retinopathy lesions and grading using transfer learning. J. Pers. Med. 12:1454. 10.3390/jpm12091454 36143239 PMC9501488

[B46] Shrager Y. Kirwan C. B. Squire L. R. (2008). Neural basis of the cognitive map: Path integration does not require hippocampus or entorhinal cortex. Proc. Nat. Acad. Sci. U. S. A. 105, 12034–12038. 10.1073/pnas.0805414105 18687893 PMC2575247

[B47] Sisodia P. S. Ameta G. K. Kumar Y. Chaplot N. (2023). A review of deep transfer learning approaches for class-wise prediction of Alzheimer's disease using MRI images. Arch. Comp. Methods Eng. 30, 2409–2429. 10.1007/s11831-022-09870-0

[B48] Soliman S. A. El-Dahshan E.-S. A. Salem A.-B. M. (2022). “Deep learning 3D convolutional neural networks for predicting Alzheimer's disease (ALD),” in New Approaches for Multidimensional Signal Processing: Proceedings of International Workshop, NAMSP 2021 (Springer), 151–162.

[B49] Sorour S. E. Abd El-Mageed A. A. Albarrak K. M. Alnaim A. K. Wafa A. A. El-Shafeiy E.. (2024). Classification of Alzheimer's disease using MRI data based on deep learning techniques. J. King Saud Univ. 36:101940. 10.1016/j.jksuci.2024.101940

[B50] Stevenson-Hoare J. Heslegrave A. Leonenko G. Fathalla D. Bellou E. Luckcuck L.. (2023). Plasma biomarkers and genetics in the diagnosis and prediction of Alzheimer's disease. Brain 146, 690–699. 10.1093/brain/awac128 35383826 PMC9924904

[B51] Tanveer M. Richhariya B. Khan R. U. Rashid A. H. Khanna P. Prasad M.. (2020). Machine learning techniques for the diagnosis of Alzheimer's disease: a review. ACM Transact. Multim. Comp. Commun. Appl. 16, 1–35. 10.1145/3344998

[B52] Techa C. Ridouani M. Hassouni L. Anoun H. (2022). “Alzheimer's disease multiclass classification model based on CNN and StackNet using brain MRI data,” in International Conference on Advanced Intelligent Systems and Informatics (Springer), 248–259.

[B53] Wen J. Thibeau-Sutre E. Diaz-Melo M. Samper-González J. Routier A. Bottani S.. (2020). Convolutional neural networks for classification of Alzheimer's disease: overview and reproducible evaluation. Med. Image Anal. 63:101694. 10.1016/j.media.2020.101694 32417716

[B54] Zhang F. Pan B. Shao P. Liu P. Alzheimer's Disease Neuroimaging Initiative; Australian Imaging Biomarkers Lifestyle flagship study of ageing Shen S.. (2022). A single model deep learning approach for Alzheimer's disease diagnosis. Neuroscience 491, 200–214. 10.1016/j.neuroscience.2022.03.026 35398507

